# Performance evaluation of a preclinical SPECT/CT system for multi-animal and multi-isotope quantitative experiments

**DOI:** 10.1038/s41598-022-21687-2

**Published:** 2022-10-28

**Authors:** Elena Prieto, Leticia Irazola, María Collantes, Margarita Ecay, Teresa Cuenca, Josep Mª Martí-Climent, Iván Peñuelas

**Affiliations:** 1 Medical Physics, Clínica Universidad de Navarra, Pamplona, Spain; 2 UNIMTRA, Clínica Universidad de Navarra, Pamplona, Spain; 3 Department of Nuclear Medicine, Clínica Universidad de Navarra, Pamplona, Spain; 4 IdisNA, Instituto de Investigación Sanitaria de Navarra, Pamplona, Spain

**Keywords:** Molecular medicine, Physics

## Abstract

The aim was to study the performance of the U-SPECT^6^/CT E-class system for preclinical imaging, to later demonstrate the viability of simultaneous multi-animal and multi-isotope imaging with reliable quantitative accuracy. The performance of the SPECT was evaluated for two collimators dedicated for mouse (UHS-M) and rat imaging (UHR-RM) in terms of sensitivity, energy resolution, uniformity and spatial resolution. Point sources, hot‑rod and uniform phantoms were scanned, and additional tests were carried out to evaluate singular settings such as simultaneous multi-isotope acquisition and imaging with a multi-bed system. For in-vivo evaluation, simultaneous triple-isotope and multi-animal studies were performed on mice. Sensitivity for ^99m^Tc was 2370 cps/MBq for the UHS-M collimator and 493 cps/MBq for the UHR-RM. Rods of 0.6 mm and 0.9 mm were discernible with the UHS-M and UHR-RM collimators respectively, with optimized reconstruction. Uniformity in low counting conditions has proven to be poor (> 75%). Multi-isotope and multi-bed phantom acquisitions demonstrated accurate quantification. In mice, simultaneous multi-isotope imaging provided the separate distribution of 3 tracers and image quality of the multi-mouse bone scan was adequate. The U-SPECT^6^/CT E-class has shown good sensitivity and spatial resolution. This system provides quantitative images with suitable image quality for multi-mouse and multi-isotope acquisitions.

## Introduction

Molecular imaging is a non-invasive and quantitative technique that provides functional information on a molecular level in small living animals. Such images and quantitative data obtained in animal models of human diseases^1,2^ allow researchers to investigate the onset and progression of diseases, to assess the biologic relevance of drug candidates and to monitor the efficacy of new therapeutic pharmaceuticals^3^.

Due to their relevant characteristics, Single Photon Emission Tomography (SPECT) and Positron Emission Tomography (PET) have been the most widely used molecular imaging techniques^4^ over the past two decades. Both PET and SPECT preclinical systems can provide 3D distribution of radiolabeled molecules with high sensitivity and spatial resolution. However, preclinical SPECT has proved to have several advantages, as submillimeter resolutions can be achieved. Moreover, the radionuclides used are more readily available, usually have longer half-lives, and are cheaper, thus facilitating their use in preclinical research^5^. Besides, gamma radionuclides of different energies offer the unique opportunity of the simultaneous imaging of different molecular targets in one single experiment^4^. Furthermore, the combined use of SPECT and Computerized Tomography (CT) in a SPECT/CT system helps to define the anatomic context of biochemical processes and improves the quantitative accuracy of the SPECT data^3^.

We have herein studied the performance of the U-SPECT^6^ E-class (MILabs, The Netherlands) with two stationary detectors and a multipinhole collimator, which might be changed for different animal sizes and image quality requirements^6^. The most important characteristics of the system, such as sensitivity, spatial resolution and contrast-to-noise ratio, have already been published for a set of collimators and for technetium-99 m (^99m^Tc), the most widely used gamma radioisotope^6,7^. However, a complete evaluation including additional collimators as well as results for radiotracers different from ^99m^Tc has not been published yet. Besides, we aimed to evaluate its performance for singular settings that, to the best of our knowledge, have not been previously assessed for this system. Particularly, we evaluated acquisition with the presence of disturbing radioactive sources in the surrounding of the system, simultaneous acquisition of different radionuclides which might allow the tracking of different tracers, or multi-animal imaging in a single imaging session, which might increase throughput and efficient tracer use in comparison to single animal imaging. All these settings should be tested to ensure image quality and preservation of quantification accuracy.

## Materials and methods

### System description

The U-SPECT^6^/CT E-class system (MILabs, Utrecht, The Netherlands) is an imaging device for small animals that combines a SPECT with a CT. The U-SPECT^6^ design from MiLabs uses rectangular scintillation detectors that surround a cylindrical multi-pinhole collimator. This design works in stationary mode and does not use angular steps to acquire the images. The standard version consists of three rectangular detectors arranged in a triangle which fully surround the collimator, while the E-class does not have the bottom detector^6^. Each detector has a 9.5-mm thick NaI(Tl) crystal with an active area of 50.8 × 38.1 cm^2^ optically coupled through a light guide with 55 photomultiplier tubes (PMTs)^5,8^. The multi-pinhole cylindrical collimator is exchangeable and, at our institution, two tungsten collimators are available, both with a set of 75 1.0-mm-diameter pinholes although, as the E-class does not present the bottom detector, only the upper pinholes contribute to the acquisition process. The Ultra-High Sensitivity Mouse collimator (UHS-M) is designed for mouse imaging, has a 44-mm diameter bore and is focused on a central field of view (FOV) of 7 mm axial length and 12 mm transaxial diameter^6^. The Ultra-High Resolution Rat and Mouse collimator (UHR-RM) allows rat imaging as well as up to four simultaneous multi-mouse acquisitions with a 98 mm diameter bore and a field of view of 28-mm in the diameter and 18 mm long^7^. Despite the limited FOV of both collimators, the system is able to scan larger volumes than the focused FOV by automatically translating the animal bed into the three spatial dimensions through the collimator^9^. The integrated webcams or the x-ray system provide animal location images to enable this process. The user defines the desired volume of interest to be scanned with the help of sliders, and the software calculates the necessary sequence of bed positions to fully cover the region^10^.

A dedicated bed with a half-cylindrical shape is provided for each collimator. Besides, Milabs provides a multi-mouse bed, with the same size as the rat bed but with plastic septa that divide the chamber into 4 sectors to house four mice. The image quality for this setting is not straightforward and should be characterized.

The system allows list-mode acquisition, storing energy and position of each detected photon, to enable the selection of energy windows during reconstruction. Therefore, the simultaneous acquisition of different isotopes is feasible. In addition, lower and upper windows can be selected for each photopeak for scatter correction (triple-energy-window, TEW).

The U-CT integrated in the system is mounted in-line with the SPECT in the rear position. It consists of a micro-focus X-ray tube source with a voltage range of 20 to 65 kV and a digital flat surface X-ray detector scanning in cone-beam geometry. The maximum scanning area is determined by a diameter of 75 mm and a length of 210 mm, with a minimum voxel size of 30 μm.

### Data acquisition and processing

Point sources and phantoms were scanned to characterize the system, while several in vivo experiments in mice were carried out to optimize the acquisition and reconstruction in real situations. For the phantoms, animal imaging conditions in terms of concentrations and acquisition times were mimicked. To this aim, animal studies performed with ^99m^Tc at our institution in the first year of use of the system were reviewed. It should be noted that the maximum administered preclinical activity is usually limited because the intravenous administration volume is restricted at most to 125 μL for mice and 1.25 mL for rats^11^. The mice used in these studies typically weighed 20 g and their mean administered activity was 37 MBq, representing a ^99m^Tc concentration of 1.85 MBq/mL. For rats, typical animal weight was 250 g and mean administered activity was 185 MBq, representing a ^99m^Tc concentration of 0.74 MBq/mL.

All SPECT images (phantoms and mice) were acquired in list mode for either 15 or 20 min. CT was acquired using the default protocols provided by the manufacturer (50 kV—0.43 mA for the UHS-M and 53 kV—0.33 mA for the UHR-RM).

Unless otherwise stated, all images were reconstructed using the standard parameters recommended by the manufacturer for animal imaging. For each photopeak, the window width was set to 20% for all energies except for ^125^I, for which a 100% width was used to cover the whole photopeak. For the radionuclides with several significant photopeaks, all of them were considered and summed. Pixel-based similarity-regulated ordered subsets expectation maximization (SROSEM) algorithm^12^ was used with 4 iterations and 128 subsets. The voxel size and the Gaussian filter width were adapted for each collimator: a 0.4-mm voxel size and a filter width of 0.7 mm for the UHS-M collimator and a 0.8-mm voxel size and a filter width of 1.2 mm for the UHR-RM collimator. Corrections for decay, scatter (TEW method) and attenuation were included. The attenuation map was obtained from the CT acquisition and applied after sampling of the SPECT images to the CT matrix (voxel size 80 μm for UHS-M and 160 μm for UHR-RM). When all corrections are included and a calibration factor is applied, the system provides quantitative images of radioactive concentration^13^. The software employed for the visualization and quantification of the SPECT images was PMOD (PMOD Technologies Ltd., Adliswil, Switzerland).

### Background and sensitivity

Sensitivity was examined for the two available collimators and for the following isotopes: gallium-67 (^67^Ga), iodine-123 (^123^I), iodine-125 (^125^I) lutetium-177 (^177^Lu) and technetium (^99m^Tc). A point source containing 1 to 10 MBq in approximately 0.01 mL was prepared, placed in the tip of a 0.2-mL test tube for each radionuclide and measured in a dose calibrator (Atomlab 200). For both collimators, two 600-s acquisitions covering a single bed position were performed, one with the point source centered in the FOV and one without activity in the field of view for background estimation. The total counts detected within the photopeaks were obtained from the log of the reconstructed images. Finally, the sensitivity (cps/MBq) was calculated as the ratio of the total net counts by the decay-corrected activity in the point source and the acquisition duration^14^.

1 $$Sensitivity \, (cps/MBq)=\frac{Source \, (counts)-Background \, (counts)}{A \left(MBq\right)\cdot {t}_{acq}(s)}$$

Background was reported as the total count rate within the photopeaks selected for each radionuclide.

### Energy resolution

Acquisitions performed for sensitivity tests were also used to analyze energy resolution of the system. For that, emission spectra were exported and each photopeak was fitted to a Gaussian function using the ImageJ fitting tool (Fiji, National Institute of Health, Bethesda, USA). The full width at half maximum (FWHM) was calculated to estimate the absolute energy. The relative energy resolution was then obtained by using Eq. (2):

2 $$Energy \, resolution \, (\mathrm{\%})=\frac{FWHM \, ({\text{keV}})}{Energy \, ({\text{keV}})} \cdot 100$$

### Count rate

The count rate as a function of activity was analyzed by using a point source with 411.8 MBq of ^99m^Tc in 0.2 mL placed on the tip of a 1.5 mL Eppendorf cup, and positioned at the center of the FOV of the UHR-RM collimator. List-mode acquisition proceeded for 48 h acquiring 96 frames of 30 min each. Photopeak counts per individual frame were then extracted from the reconstruction log file for the calculation of the count rates. Assuming linearity under low-count-rate conditions, the expected count rate was linearly extrapolated from the 5 lowest measured values^15^ and activities for which expected and measured count rates deviated more than 10% from each other were reported.

### Interference of external radioactive sources

The aim of the following experiment was to assess, in those laboratories that work with more than one molecular imaging device in the same room, the possible interference caused by the presence of an external radionuclide during SPECT acquisitions. Two different sources were prepared: a 1.5-mL test tube with 9.0 MBq of ^99m^Tc in 0.1 mL and a 1 mL syringe with 11.2 MBq of ^18^F. The ^99m^Tc source was placed in the centre of the FOV for SPECT imaging with the UHR-RM collimator while the ^18^F source was placed outside the device, at four different positions (namely left, right, front and rear), located 1 m away from the center of the SPECT gantry and at the same height as the animal bed. Five 5-min acquisitions were performed within an interval of 32 min, so ^99m^Tc decay factor between acquisitions was considered as negligible.

In order to evaluate the possible interferences, the spectrum of each individual detector was qualitatively analyzed, looking for the presence of a significant peak at 511 keV. Then, images were reconstructed with the standard reconstruction parameters (SROSEM algorithm, 128 subsets, 4 iterations, voxel size of 0.8 mm, filter of 1.2 mm, and decay and TEW scatter corrections while attenuation was considered negligible) using the specific calibration factor to quantify them. The counting statistics of each acquisition and the estimated activities in the reconstructed image were then evaluated.

### Spatial resolution

Two hot-rod micro-spatial resolution phantoms with 6 different size sectors were imaged to test the system resolution. The Derenzo hot-rod phantom model 850.500 (Φ = 24 mm) with capillary diameters of 1.5, 1.2, 1.0, 0.9, 0.8, and 0.7 mm was used for the evaluation of the spatial resolution using the UHR-RM collimator. A smaller model (Derenzo hot-rod 850.100, Φ = 10 mm) with capillary diameters of 0.75, 0.6, 0.5, 0.45, 0.4, and 0.35 mm was used for the UHS-M collimator. Both phantoms were imaged for three different activity concentrations of ^99m^Tc ranging from 700 to 30 MBq/mL, in order to evaluate the spatial resolution under different count rate conditions. Resembling preclinical conditions, the acquisition time was set to 20 min for all acquisitions.

Images were reconstructed using the standard reconstruction previously described (voxel size of 0.4 mm and filter width of 0.7 mm for the UHS-M collimator; voxel size of 0.8 mm and filter width of 1.2 mm for the UHR-RM collimator) and a second protocol with the minimum available voxel size (0.4 mm for the UHR-RM and 0.2 mm for the UHS-M) and a narrow filter (width of 0.45 mm for the UHR-RM and 0.24 mm for the UHS-M). In order to ease visualization, central planes covering a width of 4 mm were averaged, and the area outside the rods was masked. Spatial resolution was reported qualitatively as the smallest distinguishable rod size in the reconstructed image.

### Uniformity

Syringes and phantoms with different volume were prepared, all filled with typical ^99m^Tc concentration for animal imaging (1.85 MBq/mL for UHS-M and 0.74 MBq/mL for UHR-RM), in order to assess system uniformity under realistic preclinical conditions The wider source scanned in each collimator was designed to cover almost the whole available FOV. The activities, volumes and sizes of each source are presented in Table 1. The scanning SPECT time was set to 15 min to cover the whole source. Images were then reconstructed using our standard protocol for animal studies.

**Table 1:** Description of the geometry of the sources used for uniformity evaluation.

Setting	Collimator	Phantom					Bed positions	Quantification VOI size (mm)
		Container description	Volume (mL)	Dimensions (mm)	Activity (MBq)	Concentration (MBq/mL)		
#1	UHS-M	12 mL syringe	3	φ = 16 L = 16	5.5	1.85	19	φ = 10 L = 8
#2	UHS-M	20 mL syringe	20	φ = 19 L = 70	41	1.85	46	φ = 12 L = 24
#3	UHR-RM	30 mL syringe	12	φ = 22 L = 32	10.5	0.74	7	φ = 12 L = 16
#4	UHR-RM	250 mL bottle	250	φ = 58 L = 107	186.5	0.74	28	φ = 40 L = 48

Cylindrical volumes of interest (VOIs) were drawn centrally in each phantom (Table 1) and quantified using PMOD software. The system uniformity was calculated [Eq. (3)] based on the recommendations made by the National Electrical Manufacturers Association (NEMA).

3 $$Uniformity \, (\mathrm{\%})=100\cdot \frac{(max-min)}{(max+min)}$$

where max and min represent the maximum and minimum voxel values within the VOI.

### Multi-isotope acquisitions

A 12-mL syringe was filled with 5 mL of a solution containing equal activity concentration of ^99m^Tc and ^67^Ga (1.85 MBq/mL). Two additional syringes, each containing either ^99m^Tc or ^67^Ga with the same volume and concentration, were also prepared as reference.

The three syringes were individually imaged using the UHS-M collimator for 15 min, covering 26 bed positions. Images were then reconstructed using our standard protocol for the UHS-M collimator (SROSEM algorithm, 4 iterations and 128 subsets, 0.4-mm voxel size and filter of 0.7-mm). As a first step, visual analysis of the spectrum was conducted in the dual isotope image and compared to the single isotope acquisitions. Then, acquisitions were reconstructed using a window width of 20% for each photopeak (140 keV for ^99m^Tc, and 93, 184 and 300 keV for ^67^Ga) using the standard reconstruction. The radioactive concentration was separately quantified over ^99m^Tc- and ^67^Ga-reconstructed images, and results compared to expected concentration values. The quantification of that radioactive concentration was considered to be correct if the values deviated less than 10% from what was expected. Differences with the reference single-isotope images were also analyzed.

### Multi-bed acquisitions

In order to evaluate the performance of the U-SPECT^6^ system with the multi-mouse bed device, which enables that allows the simultaneous measurement of 4 mice within the UHR-RM collimator, three different experiments were conducted. Apart from the visual analysis, these three experiments were analyzed for their quantitative accuracy. To this aim, the radioactive concentration was examined in the reconstructed images and the results were compared to the expected concentration values.

### Uniformity

Four 20 ml syringes (φ = 22 mm) were filled up to 9 mL with the standard ^99m^Tc concentration of 1.85 MBq/mL. The scanning SPECT time was set to 15 min. Images were then reconstructed using our standard protocol for animal studies. The cylindrical volumes of interest (see Table 1) were drawn centrally in each of the four syringes and quantified using PMOD. Uniformity was calculated following Eq. (3).

### Detectability

In order to evaluate the performance of the multi-bed set-up for the discrimination of small sources, a phantom consisting of two capillarity wires forming a sinus shape (provided by the manufacturer for collinearity evaluation) was filled with the standard concentration of 1.85 MBq/mL of ^99m^Tc. First, the phantom was imaged alone, as reference, and then, three of the four syringes prepared for uniformity (1.85 MBq/mL) were added in the setting. The uniform syringes were identified and assigned to a particular position and the wire phantom was moved to each of the four positions. Therefore, 4 different acquisitions were performed and compared to the results when the capillary was scanned without any other source in the gantry. The duration of the first scan was 15 min, and the following scans lasted slightly longer to compensate for decay.

### Dual isotope

Four different 12-mL syringes (φ = 16 mm) filled up to 5 mL were used for this purpose. Two syringes contained a uniform solution of equal activity concentration of ^99m^Tc and ^67^Ga (1.85 MBq/mL), whereas the other two, used as reference data, had single ^99m^Tc and ^67^Ga activities (1.85 MBq/mL). Firstly, a visual analysis of the spectrum was conducted. The acquisitions were then reconstructed with standard parameters, and the images were visually analyzed.

### In-vivo imaging

All the procedures involving animals were carried following the regulations from the Directive of the European Parliament and of the Council (2010/63/EU) and from the Spanish Government (Real Decreto 53/2013), and in accordance with the *Animal Research: Reporting of *In Vivo* Experiments* (ARRIVE) guidelines. The study was approved by the Ethics Committee for Animal Experimentation of the University of Navarra (protocol 006-16).

Male C57BL/6J weighing between 23 and 25 g were used in this study. Mice were socially housed in individually ventilated cages (4–5 animals per cage), and lived in an air-conditioned room at 22 °dC under a 12-h light/dark cycle with access to food and tap water ad libitum during all the experiments.

### Multi-isotope study

The first animal experiment was designed to demonstrate the capacity of this device to perform simultaneous in-vivo multi-isotope studies. To this aim, a mouse was administered with three compounds labeled with different radioisotopes: 36.2 MBq of ^99m^Tc- HDP and 38.5 MBq of a protein labeled with ^67^Ga, both administered by a intravenous injection, and a drop of 35.3 MBq of ^125^I placed in a nostril. After one hour of uptake, a 60-min SPECT/CT acquisition was performed using the UHS-M collimator, and covering the whole animal with 56 bed positions. The image was reconstructed using the standard parameters.

### Multi-mouse bone scan

The second animal experiment presented here was designed to compare the images obtained with the multi-mouse bed within the UHR-RM collimator and the single animal images obtained with the mouse dedicated collimator.

Three bone scans were acquired from the same mouse (male, 25 g) on different days under the following conditions: (1) one mouse in the UHS-M collimator, (2) one mouse placed in multi-mouse bed and scanned with the UHR-RM collimator, and (3) four mice in the multi-mouse bed using the UHR-RM collimator. In all conditions, the animals were injected intravenously with ^99m^Tc-hydroxymethylene diphosphonate (^99m^Tc-HDP) (37 ± 0.1 MBq) in a tail vein and, 60 min after injection, were scanned for one hour under continuous anesthesia (isoflurane, 2%). To cover the whole animal, 54 bed positions were acquired in the UHS-M collimator and 17 bed positions in the bigger one.

The standard parameters were used in the reconstruction of the images obtained with each collimator. Additionally, the images obtained with the UHR-RM collimator were reconstructed with the standard parameters for the UHS-M collimator (0.4 mm voxel grid/0.7 mm Gaussian filter). Finally, all the images were reconstructed using the count reduction option in order to simulate both 15- and 2-min acquisitions. The images were visually analyzed by two independent operators who assessed the global image quality.

## Results

### Sensitivity

All the source acquisitions registered more than 10,000 counts within the photopeaks of interest. The background count rates and values of sensitivity obtained for all the studied radionuclides are shown in Table 2. We observed that the sensitivity obtained for the UHS-M collimator was approximately 4 to 5 times higher than for the UHR-RM.

**Table 2:** Sensitivity values obtained for punctual sources of different radionuclides using UHR-RM and UHS-M collimators.

Radionuclide	Photopeak (keV)	Background count rate (cps)	Sensitivity [cps/MBq (%)]	
			UHR-RM	UHS-M
^99m^Tc	140	64	493 (0.049%)	2370 (0.237%)
^67^Ga	93–184–300	130	638 (0.064%)	2431 (0.243%)
^123^I	159	69	352 (0.035%)	1324 (0.132%)
^177^Lu	56–113–208	121	158 (0.016%)	669 (0.067%)
^125^I	30	68	347 (0.035%)	1870 (0.187%)

### Energy resolution

Energy resolution was demonstrated to be independent of the collimator used. For photon energies between 95 and 222 keV, the energy resolution obtained remained stable below 12% FWHM, while it worsened for lower energies (Supplementary material: Figure 1). The worst energy resolution was 25.6%, for the 26 keV photopeak of ^123^I. The linear adjustment of energy resolution with respect to 1/$\sqrt{E}$ showed an R^2^ value of 0.88, while the exponential adjustment of resolution and energy fitted better (R^2^ = 0.98).

### Count rate

The last five measurements, for which linearity was assumed for extrapolation, had activities ranging from 1.6 to 2.1 MBq of ^99m^Tc (approximately 1 kcps). Then, the count rate was accurately measured, with deviations below 10%, up to 110.9 MBq (54.3 kcps). The behavior found for higher activities showed no linearity (Supplementary material: Figure 2).

### Interference of external radioactive sources

The presence of a 511-keV peak in the detected spectrum was clearly visually identified when the ^18^F source was placed either at the left or at the right side of the gantry (adjacent to the detector). Such peak was only identified in the detector adjacent to the source, as the opposite seemed to be shielded by the collimator. No influence of the external source was observed in the spectrum when the source was placed on the front or on the rear side (Supplementary material: Figure 3).

Regarding the counting statistics, the reference acquisition without disturbing source had 1.33 × 10^6^ counts in the ^99m^Tc photopeak and 7.43 × 10^4^ scatter counts estimated with the TEW method. When the ^18^F source was placed in left and right positions, scatter counts increased by 600% approximately, while the counts in the photopeak rose by 80%. However, when the ^18^F source was placed in on the rear or front sides, the counting statistics in the photopeak showed a non-significant variation, smaller than 5%. The scatter counting presented an increase of 40% when the source was in the bench side and no variation when source was in the rear part. As mentioned above, the E-class U-SPECT system does not have the bottom detector so we were unable to analyze any impact on the bottom side.

Despite the influence of the external source in the counting statistics with the scatter photons from ^18^F falling within the energy windows of interest, the measured activity in the quantified image was properly estimated with less than 4% deviation with respect to the reference one (without the disturbing source), meeting the ± 10% acceptance criterion.

### Spatial resolution

**Figure 1:**
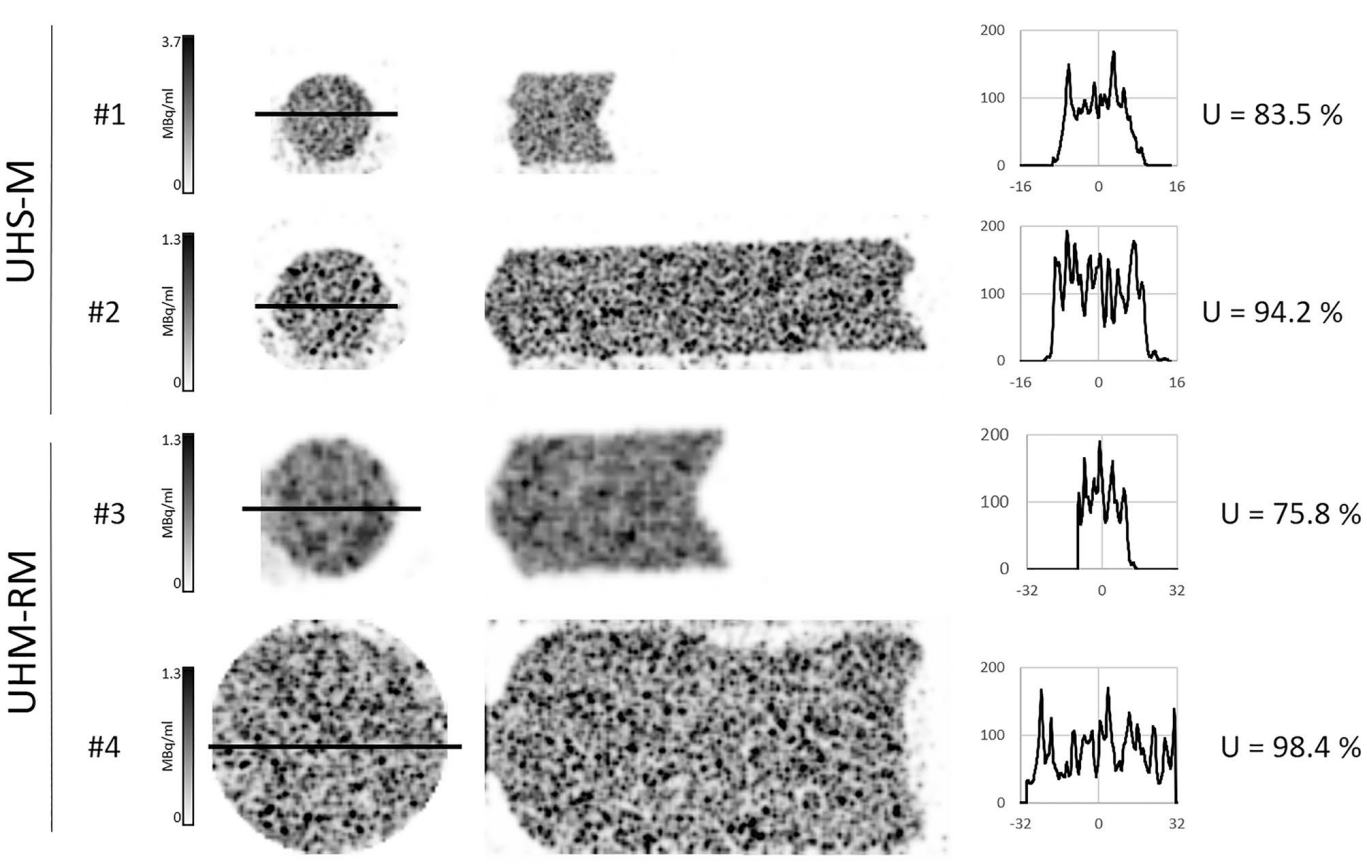
Spatial resolution values obtained with the Derenzo phantoms for the UHS-M (up) and UHR-RM (down) collimators. The image on the left shows the CT acquisition of the phantom, whereas SPECT images follow on the right. The first two images represent the same activity concentrations but different reconstruction (minimum voxel size and standard reconstruction). The color bar was adjusted according to the radioactive concentration within the phantom. Stars are used in the figure to mark the minimum distinguishable rod in each case.

Figure 1 shows in the leftmost column a CT image of the mini Derenzo hot-rod phantoms used to evaluate the system spatial resolution. For the highest concentration and the reconstruction with the smallest voxel size, the minimum visible rod size was 0.6 mm for the UHS-M and 0.9 mm and UHR-RM collimators, respectively. When the standard reconstruction was performed, values worsened up to 0.7 and 1.2 mm for UHS-M and UHR-RM collimators, respectively, but they remained constant for all the concentration values (from 700 to 30 MBq/mL).

### Uniformity

**Figure 2:**
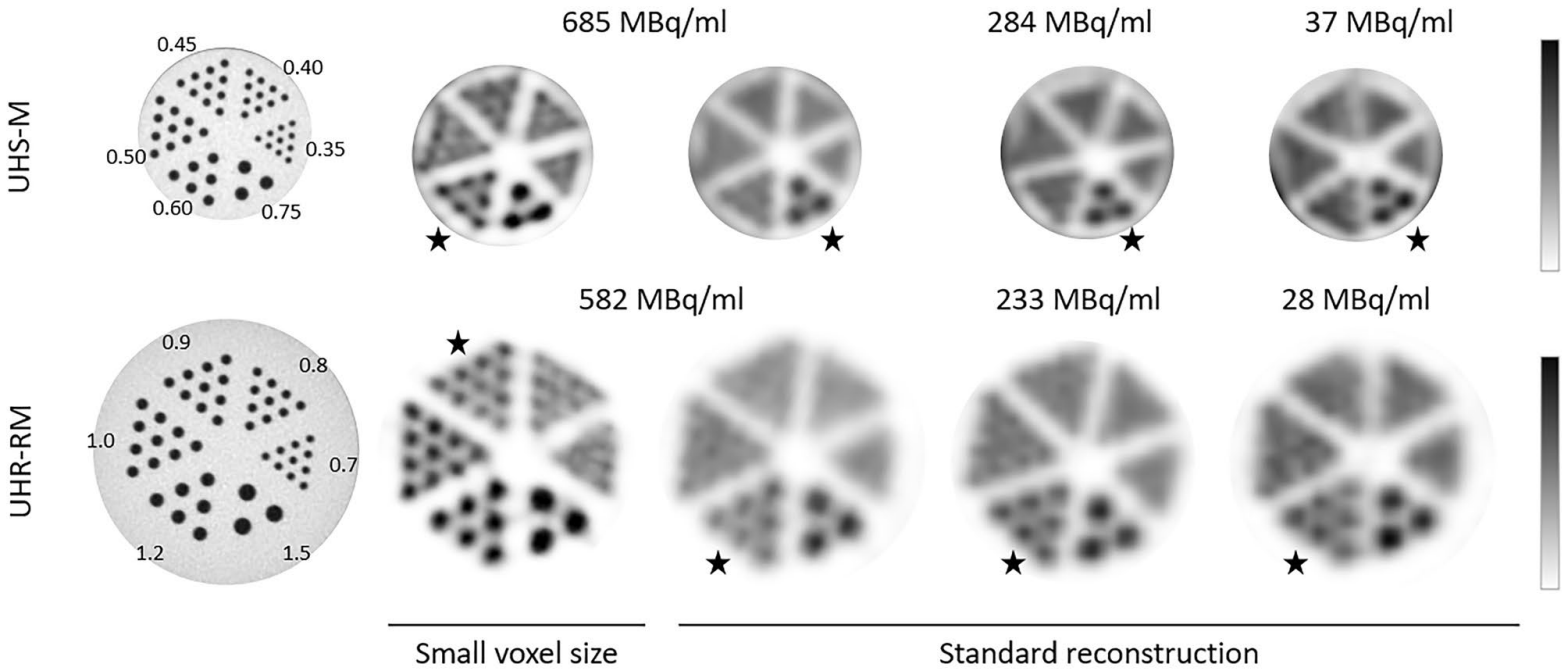
Uniformity results obtained for the UHS-M and UHR-RM collimators using different geometries (see Table 1 for detailed description).

Uniformity results, evaluated for both collimators and geometries, are presented in Fig. 2. The uniformity values were always worse for the whole field of view geometry. This behavior was also found in the profiles of the processed images (Fig. 2).

### Multi-isotope acquisition

**Figure 3:**
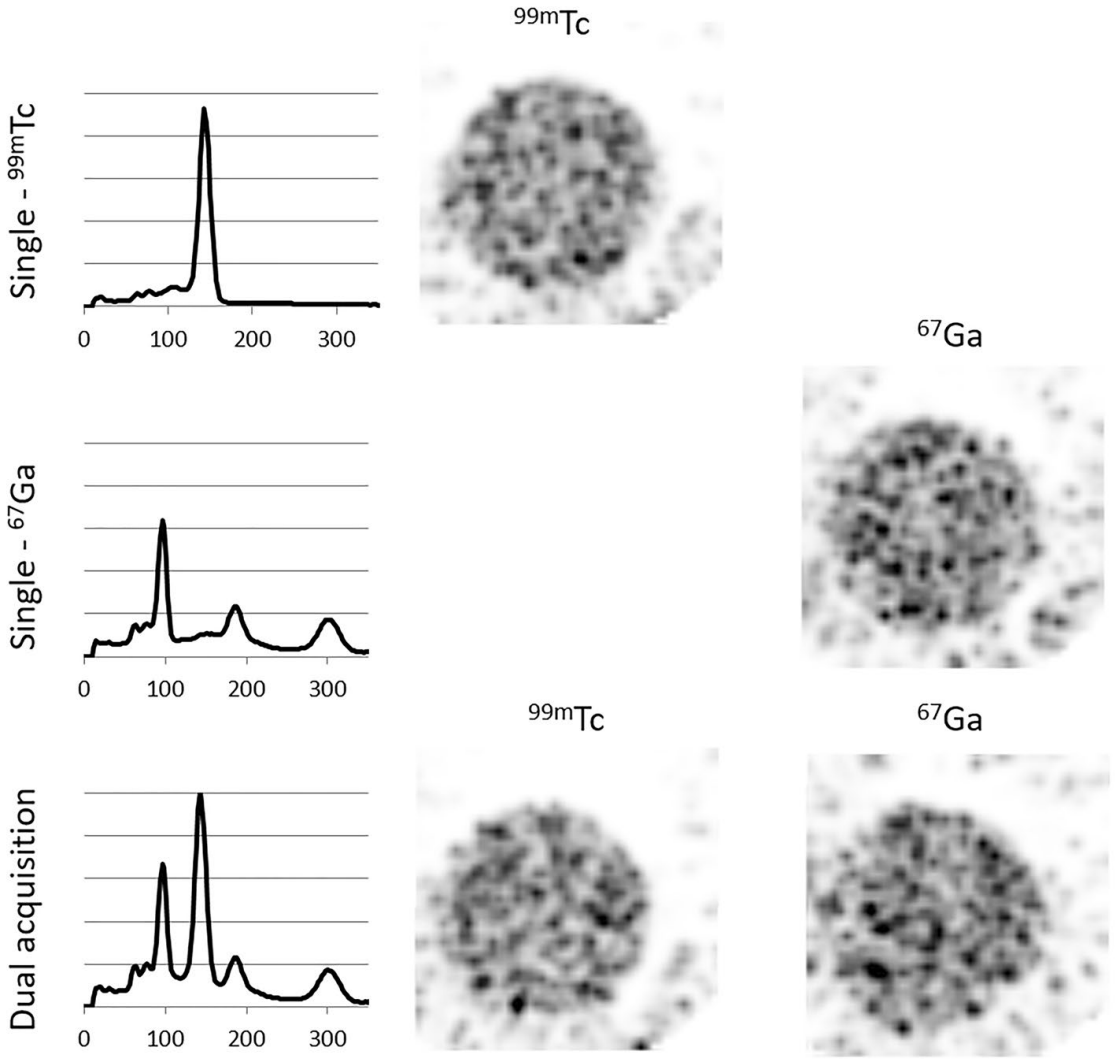
Spectra and images obtained with the single (first and second rows) and dual isotope (bottom) acquisitions with the UHS-M collimator.

Figure 3 shows the spectrum obtained with each single isotope acquisition and the spectrum of the dual acquisition. All peaks of ^67^Ga and the 140-keV peak of ^99m^Tc were clearly separated in the dual acquisition, allowing reconstruction of both radionuclides without window overlapping. However, when the dual spectrum was compared with the single spectrum, the background line was raised in the dual acquisition, an effect that was especially noticeable in the lower energy peaks (93 keV of ^67^Ga and 140 keV of ^99m^Tc).

There was no qualitative difference between the single and dual images, and they all had proper quality. However, noise could be seen outside the syringe, especially in the ^67^Ga images. The concentration of each radionuclide was accurately quantified in both single and dual acquisitions (deviation < 10%). Furthermore, the error in the ^99m^Tc quantification was − 7.2% in the single acquisition and − 8.4% in the dual acquisition, whereas for the ^67^Ga quantification it was 1.3% in the single and − 2.5% in the dual acquisition.

### Multi-bed acquisitions

#### Uniformity

**Figure 4:**
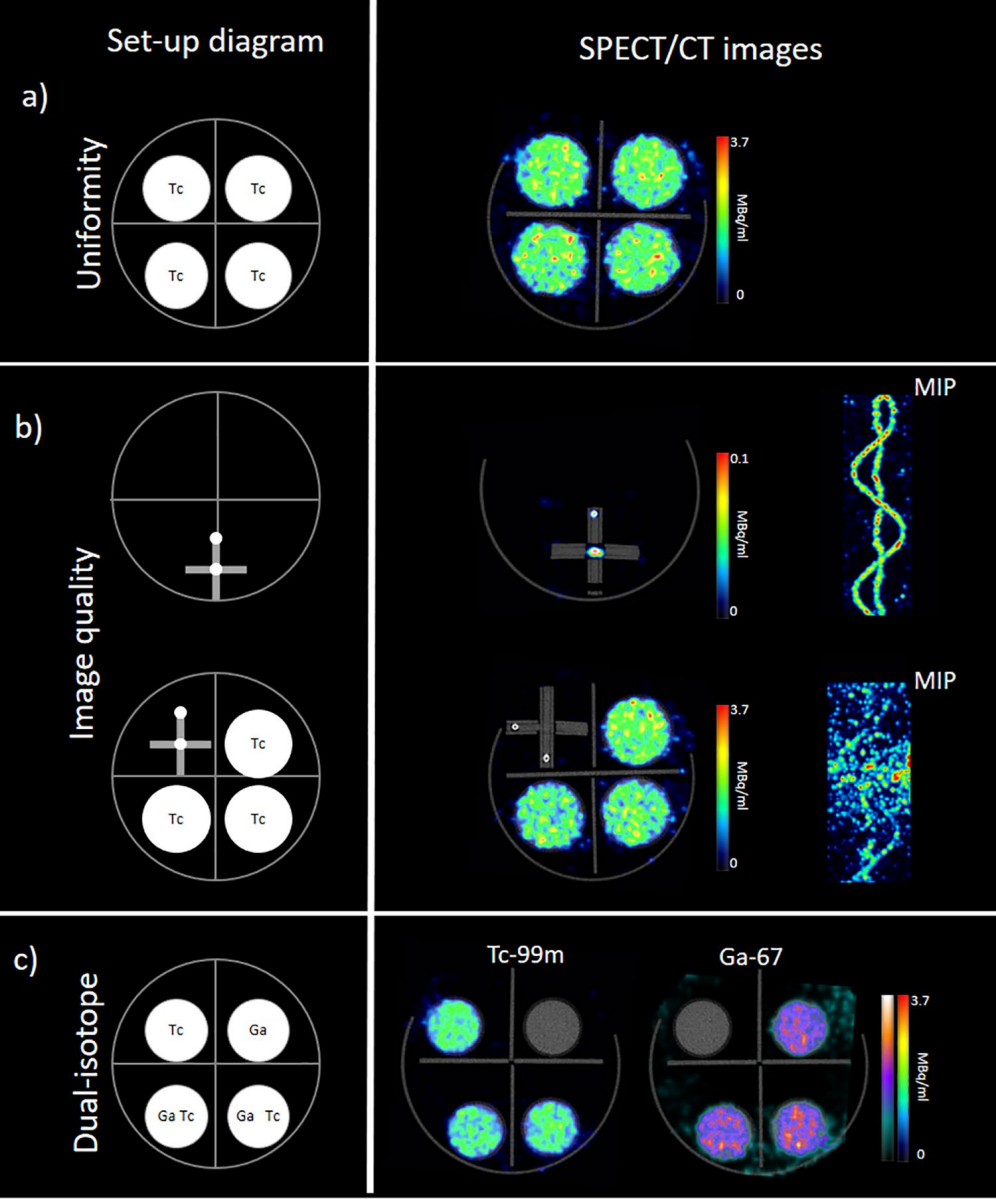
SPECT/CT images obtained with the multi-bed device and the UHR-RM collimator. The first column shows a schematic representation of the experimental set-up. (**a**) SPECT axial slice of four uniform syringes, (**b**) Sinusoidal phantom acquired alone and with three uniform syringes. Fused SPECT/CT axial slices and MIP reconstructions are shown. (**c**) Syringes with ^67^Ga and ^99m^Tc for multi-isotope performance evaluation. Fused SPECT/CT images reconstructed for each radionuclide are presented.

The uniformity values for the four syringes scanned in the multi-mouse bed were in the range [73.5–80.2], showing thus similar uniformity as the one obtained for a single uniform source in the UHR-RM collimator and better than that of an extended source covering the whole FOV. A representative image is shown in Fig. 4a. The analysis of the estimated concentration in the 4 syringes showed deviations lower than 10% with respect to the expected concentration.

#### Detectability

When the sinusoidal wire was imaged alone, it was seen as two point sources in the axial plane (Fig. 4b), and the sinusoidal shape could be clearly visualized in the MIP image. The radioactive concentration of the wire was 1.85 MBq/ml. However, due to partial volume effect, the color scale had to be saturated to 0.1 MBq/ml to be able to see the source.

When the same sinusoidal wire was scanned surrounded by three uniform syringes, the points corresponding to the wires were not visualized on the axial slices. This behavior was observed for all the four positions of the wire within the multi-bed device. In this case, the color scale was adjusted to a higher value, because syringes are wider and do not suffer the concentration underestimation caused by the limited spatial resolution. Additionally, the area of the image containing the wire was cropped to generate the MIP image (Fig. 4). In this image, the sinusoidal wires are hardly seen and the noisy MIP image does not reflect the real radioactive distribution. It should be noted that the same radioactive preparation was used, and that the acquisition time was adjusted to compensate for the decay factor and the difference in the number of explored beds. The concentration of the uniform syringes was accurately quantified in the reconstructed images (deviations < 10%).

#### Dual isotope

The reconstructed images for ^99m^Tc and ^67^Ga are presented in Fig. 4. The distribution of both isotopes is clearly differentiated. The quantification of the concentration both in the syringes with a single radionuclide and in the syringes with the mixture yielded deviations < 10%.

### In-vivo imaging

#### Multi isotope acquisition

**Figure 5:**
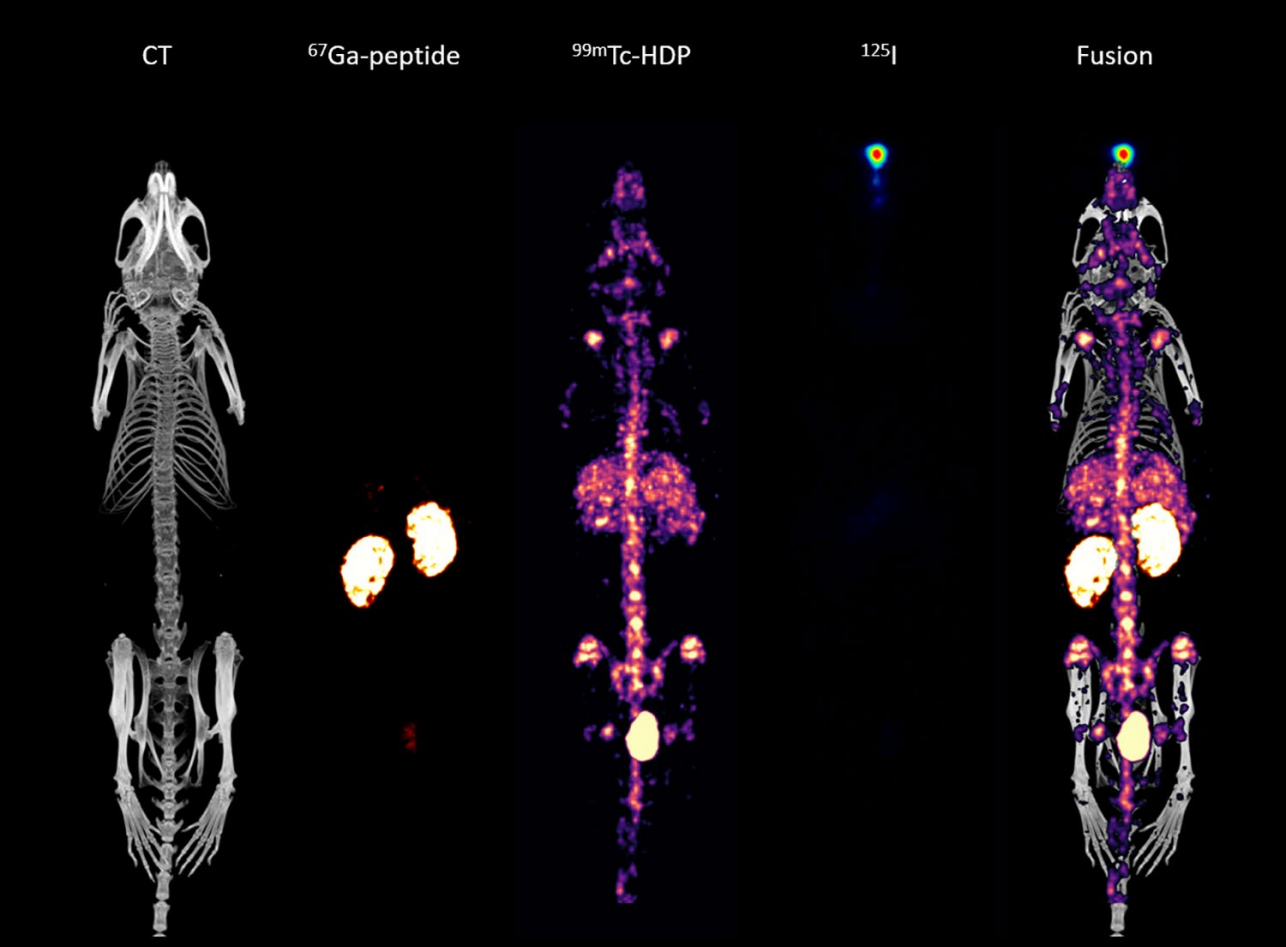
Simultaneous SPECT/CT images obtained for three different tracers labeled with ^99m^Tc, ^67^Ga and ^125^I. Separate reconstruction has been obtained for each isotope.

Multi-isotope studies in a mouse using different compounds radiolabeled with ^99m^Tc, ^67^Ga and ^125^I are shown in Fig. 5. When the reconstruction of each radionuclide was performed, three independent images were obtained, showing clearly the biodistribution of each compound without any interference between them. The image shows that the ^67^Ga-peptide is detected in the kidneys, probably due to its renal elimination, while the ^99m^Tc-HDP uptake is located in the bones, and the drop of iodine deposited in the nostril is seen in this position.

#### Multi-mouse bone scan

**Figure 6:**
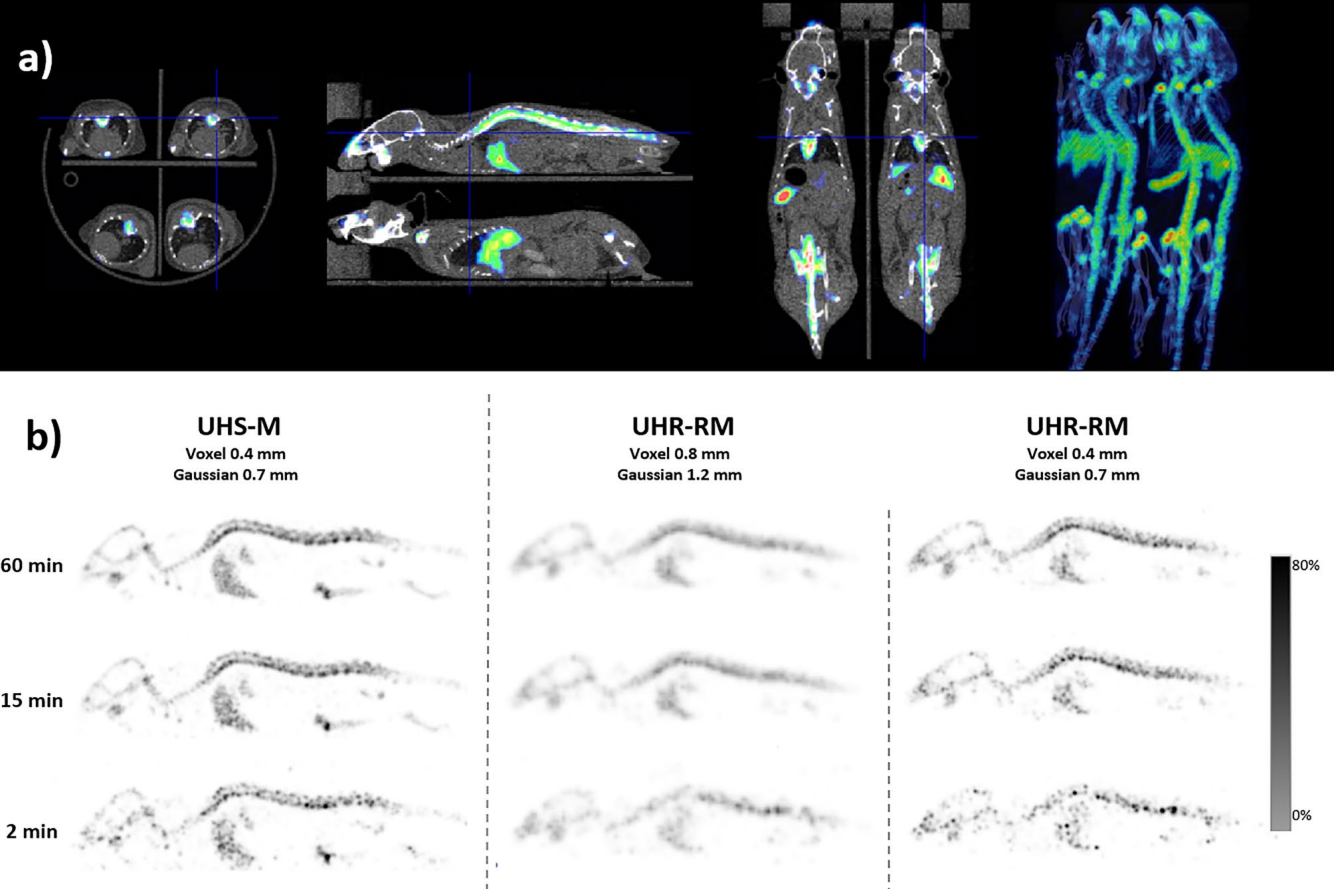
Mouse bone scan. (**a**) Axial, sagittal and coronal slices and MIP reconstruction of SPECT/CT fusion images to show multi-mouse acquisitions from four mice simultaneously. (**b**) SPECT sagittal slices of a mouse injected with ^99m^Tc-HDP using either the UHS-M or the UHR-RM collimators applying different reconstruction protocols. The ^99m^Tc HDP uptake is clearly detected in the bones (vertebrae, skull) and also in the liver.

The SPECT/CT image obtained with four mice in the multi-bed device is shown in Fig. 6a. The image quality was equivalent to the single animal acquisition, confirming that simultaneous multi-animal imaging is feasible without any loss in image quality.

Figure 6b shows the sagittal slices of the SPECT image obtained with 4 mice in the multi-bed device within the UHR-RM collimator (for comparison purposes only one animal is presented in the Figure), and reconstructed with two different sets of parameters in comparison with the acquisition in the UHS-M collimator. As expected, the optimal image quality was obtained with the UHS-M collimator, specially dedicated to mice (0.4 mm voxel size). The skull and the vertebral column can be seen, and even the bone marrow within the vertebrae is visible. When the UHR-RM collimator is used, the standard reconstruction conditions (0.8 mm voxel size) rendered very blurred images, although the uptake distribution in bones is still observable. When reconstruction parameters are modified to use a smaller voxel (0.4 mm) and a narrower filter (FWHM 0.7 mm), the spatial resolution of the images is clearly improved so that the discrimination of the vertebrae in the spine is possible. However, images are still noisy when compared with the ones obtained in the UHS-M acquisition.

Regarding the acquisition time, there were no important differences between the 60-and 15-min acquisitions in the UHS-M collimator. However, the simulated 2-min acquisition produced significantly worse images. For the UHM-RM collimator, the time was not a critical factor when images were reconstructed with 0.8-mm voxels due to the smoothness of the resultant image. However, time seems to be a critical factor when the voxel size is reduced. In this scenario, using a 60-min acquisition might be interesting to minimize the noise level to a certain extent.

## Discussion

The present study evaluated the U-SPECT^6^ E-class system. Although the most relevant performance characteristics had been previously reported, we mainly focused on the evaluation of image quality in real-life conditions with low counting statistics, in multi-animal acquisitions in a single session, and where various isotopes were studied.

The ^99m^Tc *sensitivity* values obtained here (2370 cps/MBq for UHS-M, and 493 cps/MBq for UHR-RM collimator) are similar (± 10%) to those provided by the manufacturer (2333 cps/MBq for UHS-M and 467 cps/MBq for UHR-RM collimators, respectively), and to those obtained in other publications (2054 for the UHS-M and 567 cps/MBq for UHR-RM)^6,7^.

To the best of our knowledge, the sensitivity values for other radionuclides have not been previously described in the literature for this SPECT model. The comparison with other preclinical SPECT models can be assessed, but it is clear that system characteristics are highly dependent on the configuration of the collimator. Lukas et al.^15^ estimated sensitivity for different radionuclides in the NanoSPECT/CT^PLUS^ with 4 different pinhole collimators: two dedicated to mouse imaging and other two to rat imaging. The reported sensitivity for the best collimator in terms of sensitivity (MH: mouse high resolution) for the NanoSPECT/CT^PLUS^ system was 205 cps/MBq for ^99m^Tc, 207 for ^123^I, and 59 for ^177^Lu. These values represent approximately half the sensitivity compared to the ones of the U-SPECT E-class with the UHR-RM collimator. For both systems, the lowest sensitivity was obtained with ^177^Lu. It is observed that Lukas et al*.* obtained almost the same value for ^99m^Tc and ^123^I while in our system the sensitivity of ^123^I is 25% lower than that of ^99m^Tc, despite the fact that both isotopes have approximately the same energy and a similar gamma emission branching ratio (83% for ^123^I and 89% for ^99m^Tc). However, the difference might be explained by the calibration of the dose calibrator for this isotope. According to Jacobson et al.^16^, there are substantial differences in the ^123^I calibration factor for crystal vials and for plastic recipients, as is the case of syringes and Eppendorf cups, due to the emission of K-characteristic x-rays of 27–32 keV. These authors demonstrated that, when using a dose calibrator push-button, the activity of the syringes is overestimated by 20% approximately. At our institution, the factory push-button factor was used with ^123^I (#277 in the Capintec CRC-15R), whereas Lukas et al.^15^ did not specify if the dose calibration factor had been adjusted to avoid this issue. Besides, ^67^Ga and ^125^I are two interesting isotopes in SPECT, and we have been unable to find literature determining sensitivity for them.

As *energy resolution* values had not been previously published for the U-SPECT device, we can compare them to the NanoSPECT/CT^PLUS^. The U-SPECT system has shown energy resolution below 12% for energies higher than 95 keV, slightly worse than those from the NanoSPECT/CT^PLUS^, with values below 10%^15^. Lukas et al*.*^15^ also demonstrated that for lower energies, the resolution worsened but the relationship fitted better to an exponential function rather than to the classical linear relation with 1/$\sqrt{E}$. Although we have only three energy points below 90 keV to try to derive the function (27 keV ^125^I and x-ray peaks from ^123^I and ^177^Lu), we observed an R^2^ value of 0.8827 for the relation with 1/$\sqrt{E}$ while R^2^ was 0.9833 for an exponential function, in agreement with the model proposed by Lukas et al*.*^15^.

*The count rate* measured for this device showed linear behavior up to 54 Kcps. Similarly, there is no data in the literature for our device while for the NanoSPECT/CT^PLUS^ system, the count rate linearity is preserved up to 214 kcps, being thus a significantly higher rate. Miwa et al*.*^17^ observed linearity up to 120 kcps for one bed position for the Vector system, having the same hardware as our system but with three detectors. These results indicate an adequate performance of the U-SPECT system at activity levels used in most small animal SPECT experiments. However, the count rate curves might considerably vary depending on the source extension, due to the presence of radioactive material outside the central FOV that might introduce scattering^15^. Although we have only preformed this test for the UHR-RM collimator, the behavior of the rest of collimators will be similar, but the count rate limit would be achieved at a different activity level due to the differences in sensitivity.

In this work, we have shown that this equipment is almost insensitive to the presence of ^18^F in the surroundings of the system although, to avoid *interferences*, we recommend not to place radioactive sources laterally to the system as backward detectors are only shielded with 5 mm of lead. However, on the front side, the system is shielded with 50 mm of lead except in the front collimator opening, where a 5 mm lead disc is attached to the XYZ stage. In the rear part, detectors are shielded with 50 mm of lead and the CT has an extra 5 mm lead back shield. Thus, if the U-SPECT system is placed in a room with other nuclear medicine devices, the best position would be the front or the rear part. Nevertheless, at our facility, where PET and SPECT devices are placed side to side, we have made the conservative decision not to perform acquisitions in both devices at the same time, unless it is strictly necessary.

Regarding the *spatial *resolution**,** for both collimators using the smallest voxel size, we obtained better resolution values than those reported by Janssen et al*.*^7^ (minimum discriminable rod size 1.2 mm), and Hoffman et al*.*^6^ (minimum rod size 0.75 mm). Besides, we have evaluated resolution with the standard preclinical reconstruction at our site, and realized that the optimization in terms of reconstruction protocols is of utmost importance. As the spatial resolution has proven to be independent of the count rate, at least in the studied range, a smaller voxel size is desirable at least for small animal applications where lesser focal uptakes are expected.

*A uniformity* of 75.8% for the UHS-M and 83.5% for the UHR-RM collimators was obtained, which are clearly worse than previously reported values for this system. Hoffman et al*.*^6^ presented a uniformity of 23.0% for this E-class U-SPECT combined with the UHS-M collimator (concentration 322.8 MBq/ml and non-specified acquisition time). Jansen et al*.*^7^ reported a uniformity of 55.5% for the UHR-RM, with a concentration of 30 MBq/ml and a scan time of 45 min. However, the uniformity test is highly dependent on the radioactive concentration used for the experimental setup, the acquisition time and the reconstructions parameters. The main contribution of our work is that we evaluated uniformity with the acquisition time and the concentration values representative of animal studies, which are one or two orders of magnitude lower than those presented in the studies mentioned above. The profiles presented here also show a higher variability than in previously published studies. Besides, uniformity was also tested with a radioactive source covering the whole available FOV. However, in this experiment acquisition time was fixed to 15 min because this is our current protocol for animal imaging. Hence, uniformity is observed to worsen as the number of scanned beds increases. This behavior was quite expected and suggests that acquisition time should be better adapted to the number of beds instead of using a fixed time. It should be noted that the uniformity test according to the manufacturer’s acceptance protocol was verified before this experiment (uniformity < 12% with 100 MBq of ^99m^Tc in 12 ml for UHR-RM or 3 ml for UHS-M, 1 h acquisition, and plane summation for evaluation).

As mentioned above, one of the main advantages of SPECT technique is that it allows the researchers to employ dual or even triple isotope imaging^18^. Simultaneous *dual-isotope* as compared to sequential scans of two tracers requires less acquisition time and might reduce the number of required animals while producing perfectly registered images in space and time^19^. Furthermore, radiolabeling a single chemical entity using different radionuclides can provide very valuable information, especially in fields such as nanotechnology, where both the nanoparticle scaffold and its cargo could be followed in vivo independently. However, the image quality and the quantitative accuracy might be affected by the crosstalk effect, which consists of the detection of high-energy scattered photons originated from one radioisotope in a lower energy window from the other radionuclide. This effect might be mitigated by the TEW technique available for scatter correction. In our experiment, we observed that quantification errors were lower than 10%. Besides, although Lukas et al*.*^15^ described an increase in the background noise in dual isotope acquisition, we have not observed this degradation. These authors demonstrated that simultaneous multi-isotope acquisitions especially degrade spatial resolution and image quality when radionuclide solutions are spatially superimposed. Therefore, our experiment mimics the worst scenario with overlapping spatial distribution. Simultaneous multi-isotope acquisition has also been tested in-vivo, to observe the biodistribution of two different tracers together with the deposition of a drop of a third compound. Images have shown the distribution of each tracer without interferences between them. Besides, as demonstrated with the experiments using syringes, mice images of each tracer can be accurately quantified.

The *multi-bed setup *allows the simultaneous imaging of 4 mice. At least, at our institution, this experimental setup has proven to be the most frequent and practical one, as most experimental designs require several animals for a single condition to enable the researchers to draw conclusions. Besides, tracer labeling might be a complex and expensive procedure and the product might be unstable. For this reason, the administration to several animal at the same time using the same batch of radiolabeled compound is the most efficient manner to avoid wasting the radiolabeled tracer. However, several factors might degrade the image quality, namely, a higher count rate performance, a more scatter and attenuation fraction, and the geometrical position. Multi-mouse acquisitions have already been introduced in PET imaging^20–23^ and specific tests with phantoms and animals have demonstrated that the image quality and quantitative precision is preserved, in comparison to single animal acquisition. However, to the best of our knowledge, this is the first time that this kind of multi-animal device is evaluated for SPECT.

Four-mouse simultaneous acquisitions can only be done using the UHR-RM collimator, which offers worse spatial resolution and 4–5 times less sensitivity. Then, this apparently optimal time combination provides suboptimal image quality for small mice. Despite this, uniformity in the four positions was good, similar to the uniformity obtained with the same collimator and a single radioactive source. However, the detectability of small patterns has proven to be degraded by the presence of other sources. It should be noted that the designed experiment with the sinus wire might represent an extreme situation in terms of concentration and spatial resolution. In fact, the in-vivo experiment with 4 mice in the multi-bed device, which represents a more realistic situation than the wire experiment, produced exactly the same image as compared to the single-animal SPECT. In fact, we have frequently used this multi-bed device with satisfactory results. Therefore, the image quality degradation observed in the experimental images with phantoms and syringes should not hamper the use of this setup, but protocols should be optimized in terms of radioactive concentration and acquisition time for each experimental design to obtain the best possible images. As an example, if the investigation is based on the detection of very small structures (e.g. lymph nodes in mice), the detectability may be compromised, especially if the specificity and affinity of the radiotracer is limited for that target. In these cases we would recommend increasing the acquisition time when several animals are imaged simultaneously. This is justified by the limited sensitivity of the UHR-RM collimator and also because image quality, particularly uniformity, has been demonstrated to depend on the acquisition time per bed. Besides, the optimization of the reconstruction might be critical and the minimum voxel size combined with a suitable Gaussian filter might be the preferred option.

The major limitation of this study is that each new experimental design needs a previous evaluation of the image quality requirements to optimize the image protocols. However, the experiments conducted in the present study have not undergone such assessment, because although they have been carried out thoroughly, they have followed the most frequent protocols and imaging conditions established at our research institution the previous year.

## Conclusions

The U-SPECT^6^/CT E-class system has shown high sensitivity, especially with the UHS-M collimator, and sub-millimetric spatial resolution. We have demonstrated the suitability of this device for simultaneous multi-animal and multi-isotope SPECT acquisition, not only in controlled experiments with phantoms, but also in in vivo imaging. Furthermore, quantitative accuracy was demonstrated to be preserved in these complex settings (Supplementary figures).

## Supplementary Information

Supplementary Figures.

## Data Availability

Data described in the manuscript, including all relevant images, are available from the corresponding author on reasonable request.
