# Supplementary material for: Performance evaluation of a preclinical SPECT/CT system for multi-animal and multi-isotope quantitative experiments
Source: Sci Rep. 2022 Oct 28;12:18161. doi: 10.1038/s41598-022-21687-2 (PMC9616809; doi:10.1038/s41598-022-21687-2)

SUPPLEMENTARY MATERIAL

FIGURE 1: ENERGY RESOLUTION

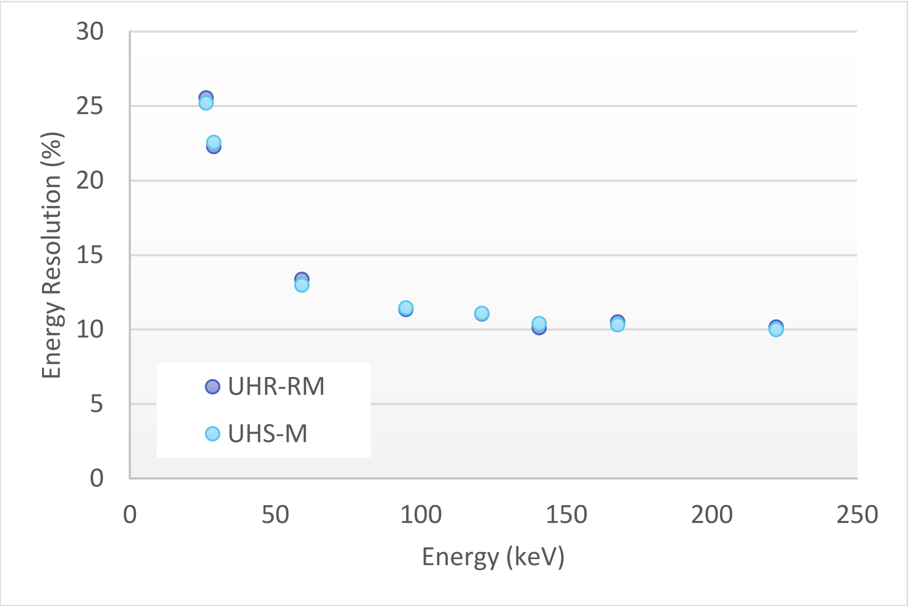

FIGURE 2: COUNT RATE

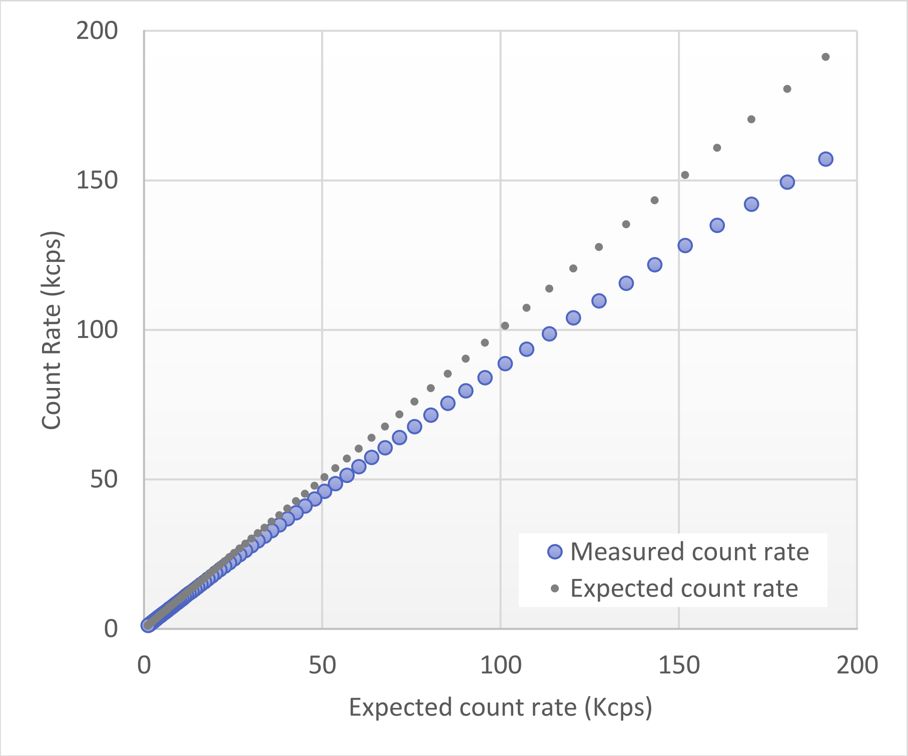

FIGURE 3: INTERFERENCE OF EXTERNAL RADIOACTIVE SOURCES

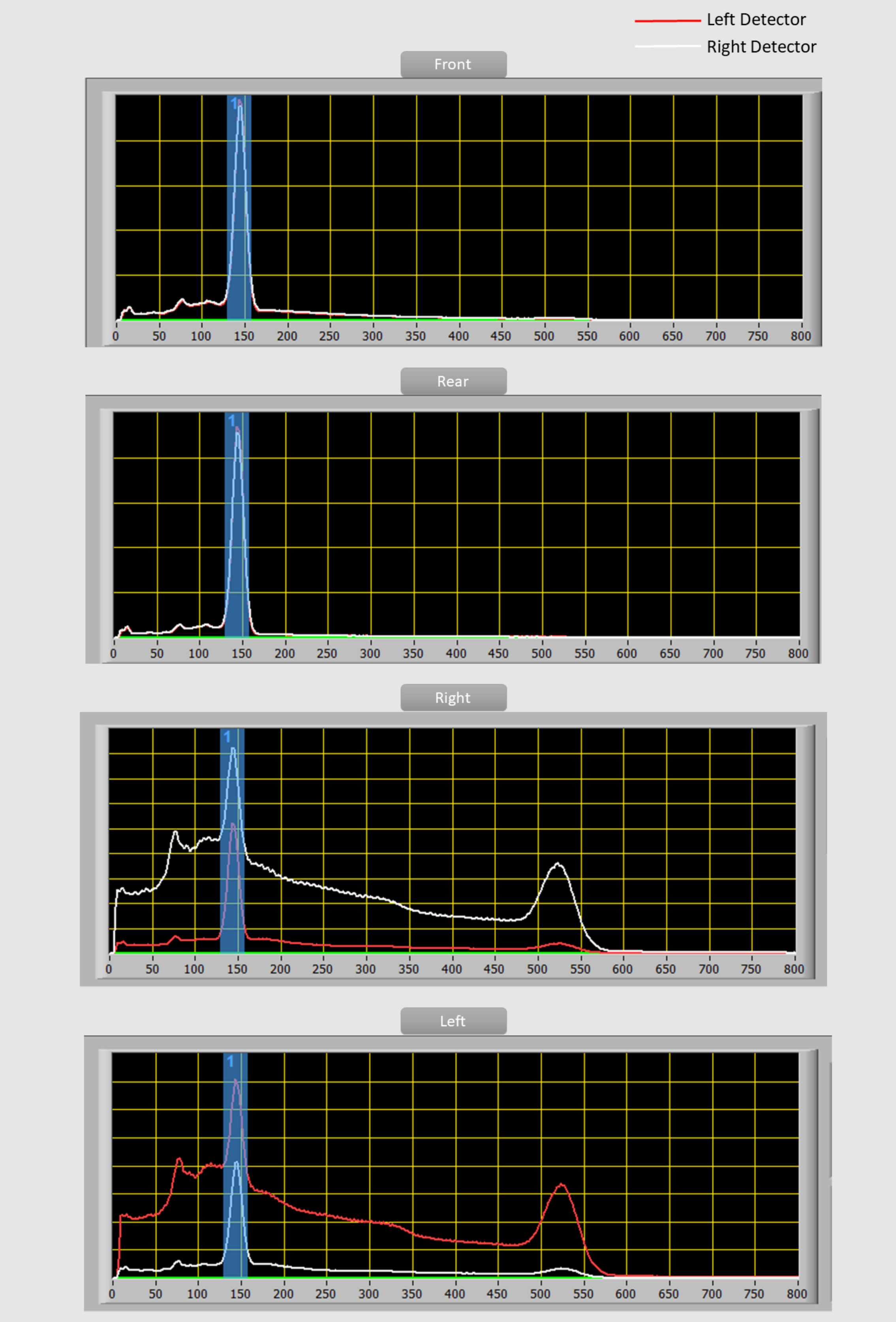

Supplement: Supplementary file 1 — Supplementary Figures. [file 41598_2022_21687_MOESM1_ESM.pdf]
